# Cardiopulmonary Bypass Surgery in a Patient with Unexpected Heparin-Induced Thrombocytopenia

**DOI:** 10.3400/avd.cr.22-00082

**Published:** 2022-12-25

**Authors:** Atsushi Miyagawa, Koichi Yuri, Mitsunori Nakano, Daigo Shinoda, Jun Makino

**Affiliations:** 1 Department of Cardiovascular Surgery, Tokyo Metropolitan Bokutoh Hospital, Tokyo, Japan; 2 Department of Critical Care Medicine, Tokyo Metropolitan Bokutoh Hospital, Tokyo, Japan

**Keywords:** heparin-induced thrombocytopenia, cardiopulmonary bypass, thrombectomy

## Abstract

We report a case of a 77-year-old woman with massive pulmonary embolism associated with heparin-induced thrombocytopenia. Before developing a pulmonary embolism, the patient underwent gastrectomy due to gastric cancer and received subcutaneous heparin calcium for deep venous thrombosis prophylaxis for 5 d. Then, thrombectomy with cardiopulmonary bypass using full heparinization was successfully performed. The patient was definitively diagnosed with this condition postoperatively, based on elevated serum antibody levels, in addition to pre- and postoperative thrombocytopenia and thrombosis. Intravenous heparin therapy was switched to argatroban. Although it is uncommon, clinicians should consider this condition in patients with a history of heparin exposure.

## Introduction

Heparin-induced thrombocytopenia (HIT) is a rare immune-mediated disorder triggered by previous heparin use. Previous studies have reported pulmonary embolism (PE) associated with HIT following cardiovascular surgery; however, few studies have reported successful cardiovascular surgery for massive PE secondary to unexpected HIT. Here, we report a case of successful surgical thrombectomy with cardiopulmonary bypass (CPB) under full heparinization for massive PE associated with HIT.

## Case Report

**Figure 1:**
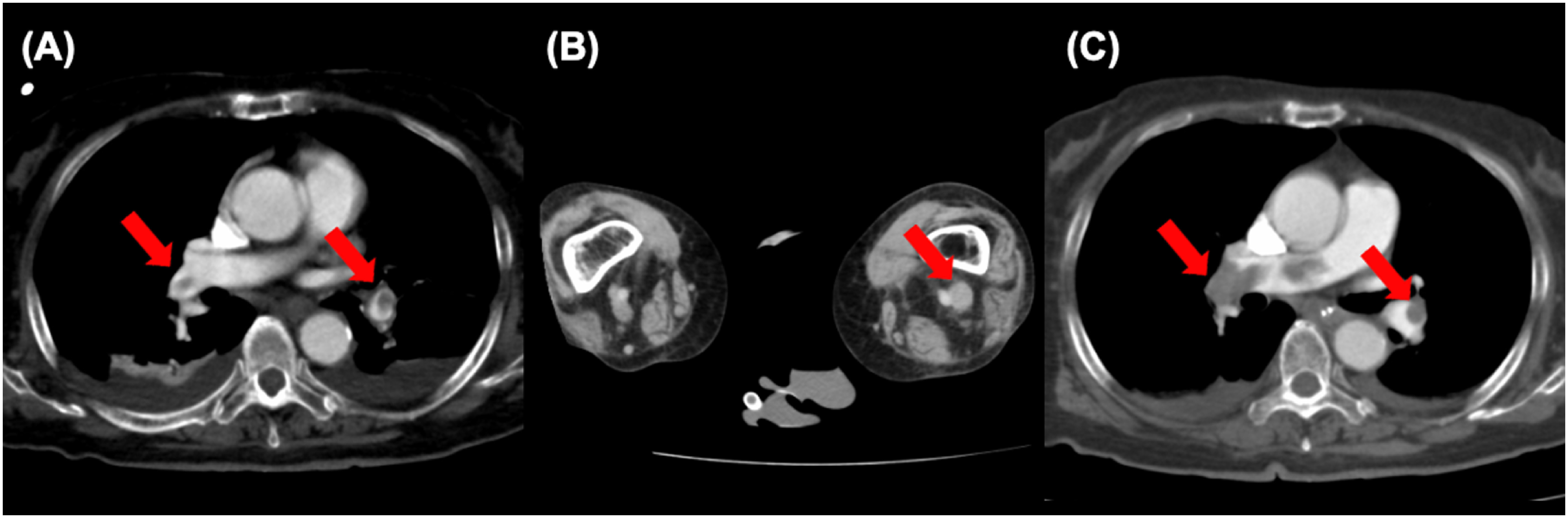
Fig. 1 (**A**) Computed tomography (CT) scan showing pulmonary embolism (PE) in the bilateral lower pulmonary arteries (PAs) on postoperative day (POD) 3. (**B**) CT scan showing deep vein thrombosis (DVT) and the popliteal venous dilation in the left lower extremity. (**C**) CT scan showing massive PE in the right main and the left lower PA on POD 5. The right PA is totally occluded by massive thrombi.

A 77-year-old woman underwent emergency distal gastrectomy for gastric perforation associated with progressive gastric cancer. A central venous catheter (CVC) was inserted preoperatively. The patient was administered 4,000 U of heparin calcium/day subcutaneously as a postoperative deep venous thrombosis (DVT) prophylaxis. The patient developed sudden tachycardia and hypoxemia on postoperative day (POD) 3. Although the patient was hemodynamically stable, poor oxygenation had developed. Contrast-enhanced chest computed tomography (CT) revealed PE in the bilateral lower pulmonary arteries (PAs) (**Fig. 1A**) and DVT in the left lower extremity (**Fig. 1B**). The d-dimer on POD 1 were not examined, but the d-dimer levels on POD 2 and 3 were 16.8 µg/mL and 88.8 µg/mL, respectively. As percutaneous heparin calcium had already been administered, and poor oxygenation had developed, continuous intravenous infusion of urokinase (20,000 U/h) and unfractionated heparin (200 U/h) was initiated.

**Figure 2:**
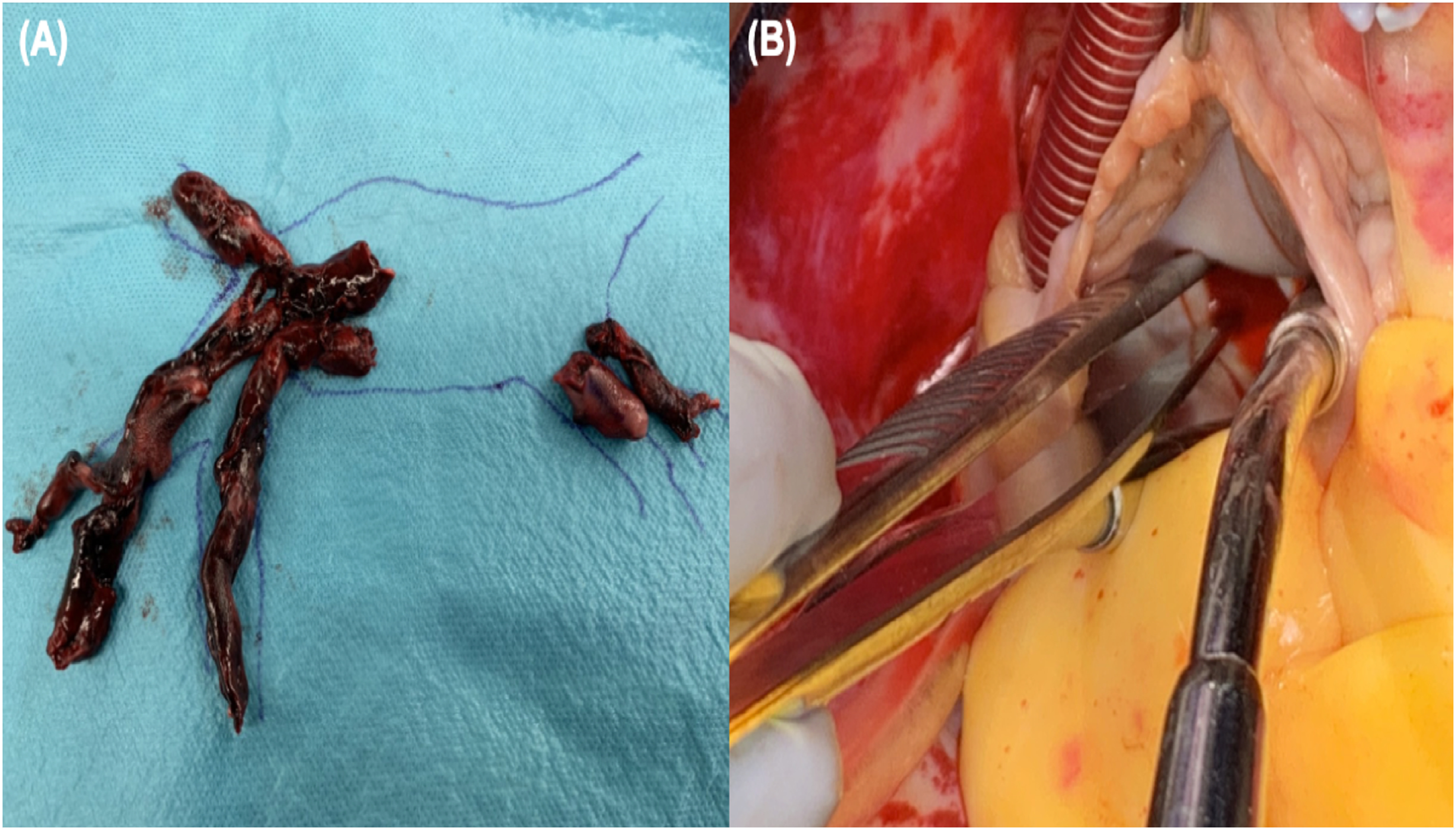
Fig. 2 (**A**) Intraoperative images showing exposed thrombi. (**B**) The patent foramen ovale is closed during the operation.

The patient presented with severe hypoxemia and hypotension on POD 5, and CT revealed massive PE with total occlusion of the right main PA and sub-occlusion of the left lower PA, despite appropriate heparinization (**Fig. 1C**). The patient presented with no unusual signs or symptoms related to the rapid response system, including high respiratory rate, arrhythmia, oliguria, and alteration of consciousness. Between the first and second PE, 960,000 U of urokinase was administered. Venoarterial extracorporeal membrane oxygenation (VA-ECMO) was initiated via the right femoral artery and vein preoperatively. Preoperative transthoracic echocardiography revealed a patent foramen ovale (PFO). Emergency thrombectomy (**Fig. 2A**) and PFO closure (**Fig. 2B**) were performed using CPB via median sternotomy. The patient received 10,000 U of unfractionated heparin. Following heparinization, an arterial cannula was inserted into the ascending aorta and a venous cannula into the superior and inferior vena cavae, and a left ventricular vent cannula was inserted through the right superior pulmonary vein. After CPB was established, VA-ECMO was switched to CPB, and systemic cooling was initiated. Aortic cross-clamping was performed after measuring a rectal temperature of 32°C. Intermittent antegrade cold crystalloid cardioplegia was administered through the aortic root. The right atrium was incised, and we observed a PFO (2 cm), which was closed using 4-0 polypropylene sutures. The pulmonary trunk and the right main PA were incised longitudinally, and thoracoscopy assisted direct thrombectomy was performed. The pulmonary trunk and right PA were closed using 4-0 polypropylene sutures with pledgets. Systemic rewarming was performed until the rectal temperature reached 35°C. Additional heparinization was not required, and the activated coagulation time (ACT) was measured at 30-min intervals during CPB. The ACT was maintained between 430 and 553 s. In view of preoperative right heart failure, we administered protamine (200 mg), and CPB was switched to VA-ECMO. The total operative, CPB, and cardiac arrest times were 289, 131, and 101 min, respectively.

**Figure 3:**
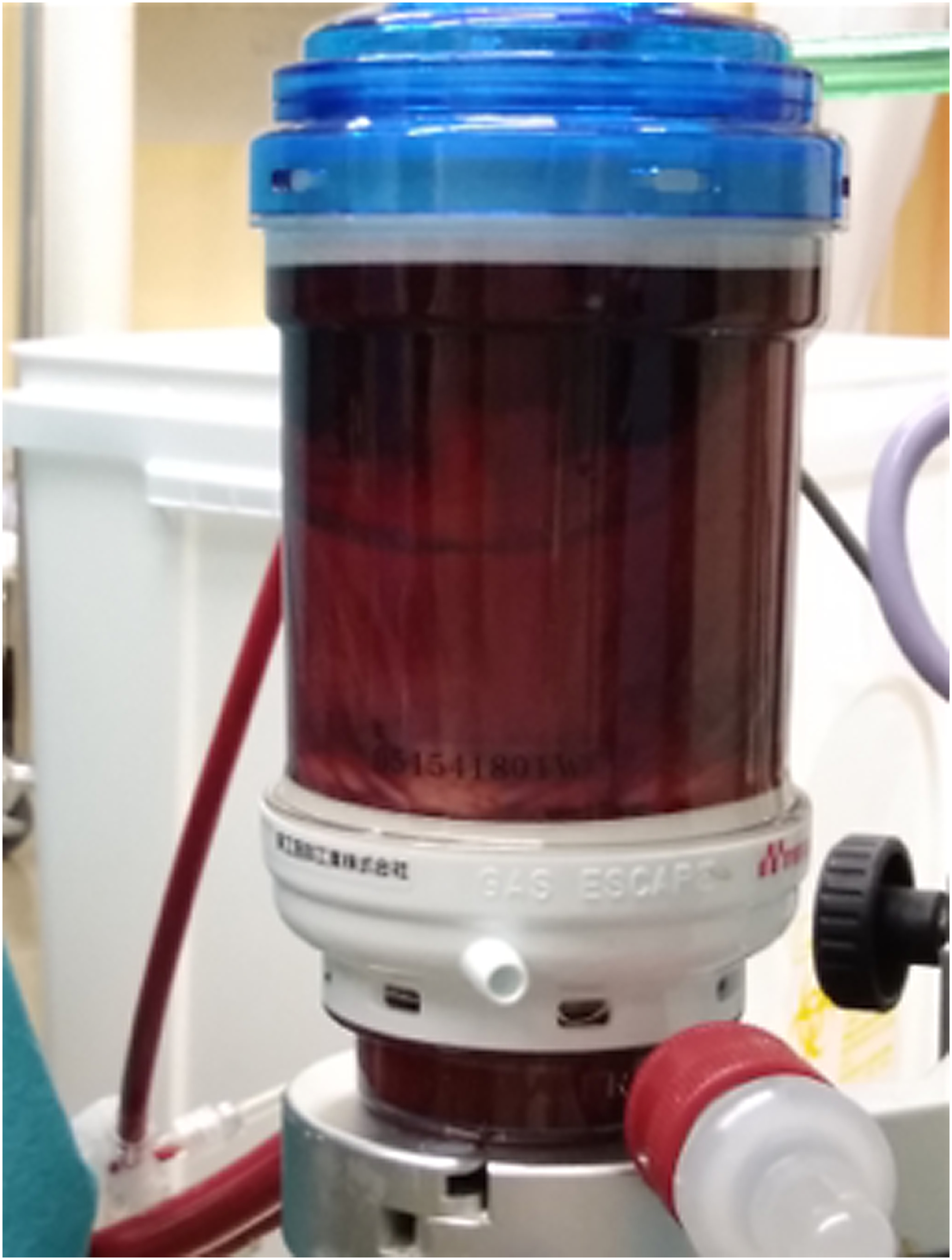
Fig. 3 Image showing the extracorporeal membrane oxygenation circuit filled with massive thrombi, the day following thrombectomy.

We observed massive thrombi surrounding the CVC following insertion of a new CVC within a few hours. The ECMO circuit was filled with massive thrombi over 24 h despite appropriate heparinization (**Fig. 3**). Although the patient was withdrawn from ECMO on POD 1 due to the above reason, thrombosis of the right femoral artery through which the ECMO cannula was inserted necessitated another thrombectomy. The platelet count before gastrectomy was 280×10^9^/L but subsequently decreased to 245×10^9^/L after surgery, and then to 52×10^9^/L 5 d later. Based on the clinical course and laboratory findings, we strongly suspected HIT in this patient. Heparin therapy was switched to argatroban, 2 d after PA thrombectomy. The serum level of antiplatelet factor 4/heparin immunoglobulin G (PF4/H) was 29.86 U/mL (reference level <1 U/mL). Laboratory tests showed negative results for thrombophilia markers such as protein C, protein S, anti-nuclear acid antibody, cardiolipin antibody β2-glycoprotein-1 complex GP1, and anticardiolipin antibody-immunoglobulin G, except for the HIT antibody. The platelet count recovered after cessation of heparin administration and initiation of argatroban therapy.

Postoperative contrast-enhanced chest CT revealed no apparent thrombosis in the PAs. The patient was weaned off of mechanical ventilation 6 d after the PA thrombectomy. Argatroban administration was continued for 10 d. The patient’s postoperative course was uneventful.

## Discussion

HIT occurs in less than 0.1%–5.0% of patients exposed to slight usage of heparin products. Onset of HIT typically varies between 5 and 10 d after heparin therapy initiation. Clinical evaluation of HIT includes determination of thrombocytopenia, timing of platelet count reduction relative to heparin administration, onset of thrombosis or other sequelae, and absence of other causes to account for thrombocytopenia. Diagnosis of HIT is based on a combination of clinical evaluation and laboratory evidence of PF4/H antibodies, both of which were used to diagnose this patient. Treatment of HIT includes cessation of heparin therapy and initiation of a non-heparin anticoagulant.^1–3)^ Our patient met all the HIT criteria and eventually was diagnosed with HIT; however, the diagnosis was delayed. Retrospectively, we should have been more careful because of the DVT and subsequent PE, despite administering standard DVT prophylaxis. However, the PE exacerbation despite appropriate heparin infusion was unusual.

An underlying malignancy, peritonitis due to gastric perforation, and major abdominal surgery triggered the primary PE in this index case. The PE was aggravated due to HIT following administration of prophylactic heparin for DVT and catheterization. We were unaware of the HIT at that point because we thought the decrease in platelet count was related to the disseminated intravascular coagulation due to the sepsis that followed gastric perforation. D-dimer levels could have been affected by factors other than PE, including the underlying malignancy and sepsis. Predicting HIT in this patient was not helped by the relatively low d-dimer levels on the second massive PE (14.3 µg/mL) in comparison to the d-dimer levels on the first PE of 88.7 µg/mL.

In view of the significant bleeding risk, thrombolytic agents were not indicated in this patient, who had a history of recent major abdominal surgery and thrombocytopenia. However, as percutaneous heparin calcium had already been administered and, consequently, the activated partial thromboplastin time was controlled well, and intubation was required due to poor oxygenation, thrombolysis was performed in addition to continuous venous heparin infusion. Although not standard, the European Society of Cardiology guidelines indicate that thrombolysis in hemodynamically stable patients with PE is acceptable.^4)^ Urokinase was the first to be chosen because it requires continuous infusion, whereas monteplase requires bolus injection. Thus, we considered it easier to adjust the amount of urokinase and minimize the risk of bleeding. However, the PE aggregated, and further treatment was needed. Surgical thrombectomy was performed, considering the patient’s significantly unstable hemodynamic status, unavailability of interventionists with expertise in catheter-assisted thrombus removal, and detection of PFO.^5,6)^

Usually, patients diagnosed with HIT after heparin exposure tend to show high morbidity and mortality rates after cardiac surgery.^3)^ Although cardiovascular surgery in patients with a history of HIT or HIT secondary to cardiovascular surgery is described in the literature,^1–3,7)^ few studies have reported surgical thrombectomy in a patient with HIT. Campisi et al. reported a case of thrombectomy of a giant left ventricular thrombus associated with HIT under heparinization for CPB.^8)^ Van Damme et al. reported thrombectomy for PE in a patient with HIT using platelet aggregation inhibitors.^9)^ However, successful thrombectomy of massive PE and PFO closure using full heparinization CPB in a patient with HIT without administration of other anticoagulants or platelet aggregation inhibitors were not described before. Fortunately, CPB was successful without intraoperative thrombosis in this case, and the patient was discharged without severe complications. We believe the surgery did not lead to immediate systemic thrombosis because of the short interval between the surgery and the PE onset, but the truth remains unknown.

Medical professionals should be aware of HIT whenever using heparin; if HIT is suspected, the platelet count should be monitored closely; heparin administration, including for extracorporeal circulation, cannulas, and catheters, should be terminated immediately; and argatroban should be administered instead. Furthermore, all medical staff in charge of the patient should be notified of the possible HIT. We have emphasized the history of HIT and the prohibition of heparin use on the top page of the patient’s electronic chart. We also provided information on the HIT in a letter to the follow-up physician. The patient’s family was told to notify the attending physicians about the risk of HIT, even if the patient was admitted to another hospital.

## Conclusion

Although HIT is a relatively uncommon disorder, clinicians should consider this condition in the differential diagnosis in patients with a history of heparin exposure. Immediate cessation of heparin administration and switching to other anticoagulants such as argatroban with close monitoring for thrombus formation are essential in patients with a high index of clinical suspicion for HIT.
